# Long-term effect of sleeve gastrectomy surgery on Hormonal Profile, Semen Parameters and sexual functions of obese infertile men; a prospective observational study

**DOI:** 10.1186/s12610-023-00191-1

**Published:** 2023-06-22

**Authors:** Tamer A. Abouelgreed, Adel Elatreisy, Ahmed F. El-sherbeiny, Mohamed A. Abdelaal, Tamer Saafan, Osama Shalkamy, Hamdy Farag, Osama M. Ghoneimy, Eman M. El-dydamony, Eman H. Ibrahim, Mohamed Amer, Khalid Kutub, Mohamed Zamra, Mohamed A. Hussein, Ayman K. Koritenah, Sherin A. Hefny

**Affiliations:** 1 Department of Urology, Faculty of Medicine, Al-Azhar University, Cairo, Egypt; 2 Department of Andrology, International Islamic Center for Population Study and Research, Al-Azhar University, Cairo, Egypt; 3 Department of Surgery, Gulf Medical University, Ajman, UAE; 4 Department of Surgery, Faculty of Medicine, Al-Azhar University, Cairo, Egypt; 5 Gulf Medical University, P.O. Box 11117, Ajman, UAE; 6 Department of Pathology, Faculty of medicine, Al-Azhar University, Cairo, Egypt; 7 Department of Dermatology and Andrology, Al-Azhar University, Cairo, Egypt; 8 Department of Urology, Al sharq hospital, Fujairah, UAE; 9 Department of Urology, AlQasemi Hospital, Sharjah, UAE; 10 Department of Surgery, Saudi German Hospital, Ajman, UAE; 11 Department of clinical Pathology, Ministry of Health and Prevention, Dubai, UAE

**Keywords:** Obesity, Semen parameters, Sleeve gastrectomy, Obésité, Paramètres du Sperme, Gastrectomie longitudinale, Sleeve Gastrectomie

## Abstract

**Background:**

The effect of bariatric surgery on impaired semen parameters, hormonal profile and sexual function remains controversial to some extent.

**The context and purpose of the study:**

To look at the long-term effects of sleeve gastrectomy on hormonal profiles, sperm parameters, and sexual function in infertile men with severe obesity. This prospective study included fifty-four obese patients with primary or secondary infertility who were scheduled for sleeve gastrectomy between February 2018 and March 2021. All participants were given a sperm analysis and a serum hormone profile before, 12, and 18 months after surgery. We used the International Index of Erectile Function questionnaire to assess sexual function.

**Results:**

There was a significant correlation between weight loss after sleeve gastrectomy and improvement in lipid profile (*p* < 0.05). No significant detectable effect of post-gastrectomy weight loss on patients with diabetes mellitus, hypertension, or obstructive sleep apnea. As regards the hormonal profile, sex hormone binding globulin, total and free testosterone improved significantly after 12- and 18-months following sleeve gastrectomy. There was a significant increase in sperm count and total sperm number during the follow-up after sleeve gastrectomy (*p* < 0.05), however, there were no significant changes in other semen parameters. Concerning sexual function, sexual desire, erectile function, and satisfaction improved significantly at 12 and 18 months after surgery.

**Conclusion:**

Weight loss through sleeve gastrectomy surgery significantly improves testosterone deficiency, sexual performance, and Sperm count in obese infertile men.

## Résumé

**Contexte:**

L’effet de la chirurgie bariatrique sur l’altération des paramètres du sperme, du profil hormonal et de la fonction sexuelle, reste controversé dans une certaine mesure. Le contexte et le but de l’étude : examiner les effets à long terme de la gastrectomie longitudinale sur les profils hormonaux, les paramètres du sperme et la fonction sexuelle chez les hommes infertiles souffrant d’obésité sévère. Cette étude prospective comprenait 54 patients obèses, atteints d’infertilité primaire ou secondaire, qui devaient subir une gastrectomie longitudinale entre février 2018 et mars 2021. Tous les participants ont eu une analyse de sperme et un profil hormonal sérique avant, puis 12 et 18 mois après la chirurgie. La fonction sexuelle a été évaluée au moyen du questionnaire de l’indice international de la fonction érectile.

**Résultats:**

Une corrélation significative était présente entre la perte de poids après la gastrectomie longitudinale et l’amélioration du profil lipidique (*p* < 0,05). Aucun effet significatif détectable de la perte de poids post-gastrectomie n’a été retrouvé chez les patients atteints de diabète, d’hypertension ou d’apnée obstructive du sommeil. En ce qui concerne le profil hormonal, la globuline liant les hormones sexuelles, la testostérone totale et la testostérone libre se sont significativement améliorées à 12 et 18 mois après la gastrectomie longitudinale. Il y eut une augmentation significative de la numération de spermatozoïdes et du nombre total de spermatozoïdes au cours du suivi après la gastrectomie longitudinale (*p* < 0,05) ; sans, toutefois, de changements significatifs pour les autres paramètres du sperme. En ce qui a concerné la fonction sexuelle, le désir sexuel, la fonction érectile et la satisfaction se sont considérablement améliorés 12 et 18 mois après la chirurgie.

**Conclusion:**

La perte de poids due à la chirurgie par gastrectomie longitudinale améliore significativement le taux de testostérone, la performance sexuelle et le nombre de spermatozoïdes chez les hommes infertiles obèses.

## Introduction

Obesity has become one of the most serious health problems worldwide. Over the past three decades, the prevalence of obesity has nearly doubled and has reached epidemic proportions, becoming a global health problem [1, 2]. Obesity in men is associated with infertility and hormonal imbalances. The explanation for this association may be related to the effect of obesity on male reproductive hormones. Significant reductions in total testosterone, free testosterone, and sex hormone-binding globulin and elevated estradiol levels in obese men have been well documented [3]. Sermondade et al., reported an association between an increased risk of infertility in overweight or obese male partners and the higher incidence of sperm abnormalities observed in these patients [4]. Weight loss following lifestyle changes has been reported to increase free and total testosterone levels while improving obesity-related metabolic comorbidities in obese men [5, 6]. Bariatric surgery is currently considered the most effective treatment for obesity. Regarding gonadal function, resolution of obesity-induced secondary hypogonadism in males was reported in 87% of patients after surgery. Reports regarding the effect of weight loss through lifestyle modifications on sperm parameters are scarce; still, it seems to improve sperm count and total sperm number, which could enhance fertility capability and decreases the time to pregnancy [7, 8]. However, the effect of bariatric surgery on semen parameters or sexual function remains controversial [9]. This prospective study aimed to investigate the long-term effects of gastric sleeve surgery on hormonal and semen parameters and sexual function in severely obese infertile men.

## Materials and methods

A total of 59 severely obese patients with primary or secondary infertility scheduled for sleeve gastric surgery were enrolled in this prospective observational study between February 2018 and March 2021. Five patients did not have long-term follow-up, and 54 patients completed the study. The study included patients who met the National Institutes of Health’s bariatric surgery criteria and were scheduled for surgery. All participants signed written informed consent to participate in this study. This protocol was approved by the Clinical Research Ethics Committee of Thumbay University Hospital (affiliated with Gulf Medical University, REC No: 308/2018). All procedures involving human participants conformed to the 1964 Declaration of Helsinki and its subsequent amendments or similar ethical standards. Exclusion criteria in our study included patients over 55 years of age and patients with renal, hepatic, gonadal, or endocrine disease or concomitant therapy that could affect sex hormone levels or semen parameters, history of malignant testicular tumors, trauma or dysplasia, and history of vascular surgery. All included patients were examined before surgery (baseline), 12 months and 18 months after gastrectomy. Parameters such as weight, height, body mass index (BMI), and obesity-related comorbidities were recorded before surgery and during follow-up. Dyslipidemia was defined as the presence of any of the following: triglycerides ≥1.7 mmol/L, high density lipoprotein cholesterol (HDLc) < 1 mmol/L, low density lipoprotein cholesterol (LDLc) > 4.14 mmol/L, or previous lipid-lowering therapy. Percent total weight loss (%TWL) was defined as preoperative weight minus follow-up weight, divided by preoperative weight and multiplied by 100 [10]. All included participants underwent semen analysis and serum hormone analysis before surgery, 12 months and 18 months after surgery. This assessment time point was chosen to ensure adequate weight loss and recovery from the initial deleterious effects of rapid weight loss on semen parameters and to examine the long-term effects of gastrectomy on semen parameters, hormonal profiles and sexual function. Semen analysis is done after 3-5 days of abstinence. Semen samples were collected in clean containers by masturbation at each study visit to the hospital campus. Samples were incubated at 37 °C and liquefied for 30 minutes prior to analysis. Analysis was performed according to the methods and reference limits of the WHO 2021 guidelines [11]. Hormonal parameters measured included sex hormone-binding globulin (SHBG), follicle-stimulating hormone (FSH), luteinizing hormone (LH), prolactin, testosterone, and estradiol. We utilized the electrochemiluminescence immunoassay (Cobas E601 analyzer; Roche Diagnostics GmbH, Mannheim, Germany) to estimate serum testosterone and prolactin. The chemiluminescent microparticle immunoassay (CMIA) (Architect; Abbott Laboratories, Chicago, IL, USA) was used for assessment of estradiol, FSH, and LH. the chemiluminescent enzyme immunoassay (Immulite 1000; Siemens Healthcare Diagnostics, Llanberis, UK) was used to measure SHBG. We calculated free testosterone by Vermeulen’s eq. [12]. Hypogonadism was defined as a total testosterone level < 9.2 nmol/L [13]. For the evaluation of Sexual function, we utilized the International Index of Erectile Function (IIEF) questionnaire to assess sexual desire, erectile, and orgasmic functions, and evaluate the patient’s satisfaction. The total possible score for the erectile function domain ranges from 1 to 30, and we classified erectile dysfunction into four categories: no erectile dysfunction (26–30), mild (17–25), moderate (11–16), and severe (6–10) [14]. Laparoscopic sleeve gastrectomy was performed on all patients by the same surgical team according to the method described by Gagner et al., [15]. The dietitian then regularly monitors the patient’s adherence to the postoperative meal plan.

## Statistics

The statistical analyses were carried out using the SPSS 28.0 software. For continuous variables, means and standard deviations (SD) were used, and absolute numbers with percentages were used for categorical variables. During the follow-up period, we used non-parametric tests (the Wilcoxon and McNemar tests) to analyze the continuous and categorical variables, respectively. Spearman correlation analysis was used to determine correlations between observed changes. *P* < 0.05 was considered significant.

## Results

A total of 54 male patients were included in the study. BMI was significantly reduced in all patients (*p* < 0.05), and the clinical data at preoperative and final follow-up are shown in Table 1. There was a significant correlation (*p* < 0.05) between weight loss after gastric sleeve surgery and improvement in dyslipidemia, HbA1c (%), triglycerides, total cholesterol, LDLc, and HDLc (Table 1). On the other hand, this study showed that weight loss after gastrectomy had no significant effect on patients with diabetes, hypertension or obstructive sleep apnea (OSA). Fasting blood glucose levels were only slightly affected by weight loss after gastrectomy (Table 1). Eighteen patients (33.3%) had preoperative male obesity secondary hypogonadism (MOSH), all of which resolved completely after surgery (Table 2). With regard to hormonal profile, SHBG, total testosterone, and free testosterone improved significantly 12 and 18 months after gastric sleeve surgery (Table 2). The preoperative mean total serum testosterone level increased from 11.72 ± 4.91 to 22.80 ± 4.80 nmol/L at 18 months postoperatively; similarly, the preoperative mean serum free testosterone level increased from 0.24 ± 0.04 to 0.38 ± 0.13/L (*p* < 0.05). The elevations in these hormone concentrations and SHGB observed after gastrectomy surgery were similar to the presence or absence of hypogonadism. At 18 months, there was a significant positive correlation between increases in total testosterone and percent of weight loss (rs 0.781; *p* = 0.004). Changes in SHBG at 18 months were also associated with overall weight loss rates (rs 0.7168; *p* = 0.013). Regarding semen parameters, initial semen analysis revealed oligospermia in 21 (38.9%) patients, asthenospermia in 13 (24.1%) patients, teratospermia in 8 (14.8%) patients, and twelve (22.2%) patients have abnormalities in more than one semen parameter. Sperm count significantly increased during the follow-up period after sleeve gastrectomy; it increased from 2.95 ± 1.14 to 9.87 ± 1.56 × 10^6^/ml, similarly; total sperm number significantly increased from 8.26 ± 2.17 to 28.82 ± 2.25 at the end of follow up (*p* < 0.05). The number of patients with oligospermia (sperm concentration below 16 million sperm per milliliter) (WHO, 2021) [11] was 14 out of 54 before sleeve gastrectomy and 9 out of 54 after a mean of 18 months follow-up after surgery (Table 2). Similarly, we observed that the number of patients with severe oligospermia (< 5 million per milliliter) was reduced from 7 out of 54 before the procedure to 2 out of 54 after eighteen months from sleeve gastrectomy (*P* < 0.01). There was no significant change in the remaining sperm parameters after surgery (Table 2). In our study, semen anti-sperm antibodies were not detected in any of the included patients throughout the study period. Regarding sexual function, there was no significant improvement in orgasmic function; instead, libido, erectile function, sexual satisfaction, and overall satisfaction increased significantly at 12 and 18 months after gastric sleeve surgery. All the results of the IIEF questionnaire are shown in Table 3.

**Table 1:** Clinical and metabolic data at preoperative time and at the end of follow-up

	Pre-operative	18 Months
Age (years)	46 ± 4.83	47.18 ± 4.82
BMI (kg/m^2^)	41.3 ± 4.1	28.5 ± 3.32 *
Diabetes mellitus, n (%)	13 (24.07)	10 (18.52)
Hypertension, n (%)	15 (27.8)	11 (20.37)
Dyslipidemia, n (%)	45 (83.3)	4 (7.4) *
OSA, n (%)	22 (40.74)	17 (31.48)
Fasting glycaemia (mmol/L)	6.29 ± 1.42	5.79 ± 2.22
HbA1c (%)	5.85 ± 0.58	5.11 ± 0.93 *
Triglycerides (mmol/L)	3.73 ± 1.32	0.99 ± 0.37 *
Total cholesterol (mmol/L)	4.88 ± 0.78	4.46 ± 0.91 *
LDLc (mmol/L	3.09 ± 0.67	2.61 ± 0.62 *
HDLc (mmol/L)	1.08 ± 0.14	1.37 ± 0.24 *

*BMI* Body mass index, *OSA* Obstructive sleep apnea, *LDLc* Low density lipoprotein cholesterol, *HDLc* High density lipoprotein cholesterol. Statistical test used: Wilcoxon test was used to identify the significance between continuous preoperative values and follow-up data. McNemar tests was used to identify the significance between nominal preoperative values and follow-up data. Data are expressed as mean ± SD or n (%)

* p < 0.05

**Table 2:** Comparison between preoperative hormonal assessment and seminogram, and follow-up values at 12- and 18-months post- Sleeve Gastrectomy Surgery

	Pre-operative	12 Months	18 Months
Total testosterone (nmol/L)	11.72 ± 4.91	20.66 ± 6.53 **	22.80 ± 4.80 **
Free testosterone (nmol/L)	0.24 ± 0.04	0.38 ± 0.13 *	0.38 ± 0.03 *
SHBG (nmol/L)	21.51 ± 11.06	40.46 ± 11.51 **	40.27 ± 13.37 **
FSH (UI/L)	4.21 ± 1.48	4.46 ± 1.00	5.25 ± 1.60 *
LH (UI/L)	2.93 ± 1.45	2.94 ± 1.91	3.21 ± 1.21
Estradiol (nmol/L)	0.11 ± 0.37	0.11 ± 0.03	0.10 ± 0.06
Prolactin (mUI/L)	223.06 ± 141.1	213.21 ± 155.03	214.24 ± 182.39
MOSH (N (%))	18 (33.3%)	0 *	0 *
**Oligospermic patients, (N (%))**	**14 (25.9%)**	10 (18.5%) **	9 (16.7%) **
**Severe oligospermic patients (< 5 × 10**^**6**^**/ml), (N (%))**	**7 (12.9%)**	2 (3.7%) **	2 (3.7%) **
Semen volume (mL)	2.8 ± 1.75	3.05 ± 1.38	2.96 ± 1.37
Semen pH	7.63 ± 0.29	7.66 ± 0.18	7.57 ± 0.10
Sperm count (× 10^6^ / mL)	2.95 ± 1.14	9.63 ± 1.28 *	9.87 ± 1.56 *
Total sperm number/ejaculate	8.26 ± 2.17	29.37 ± 2.21*	28.82 ± 2.25*
Leucocyte concentration (× 10^6^ / mL)	0.19 ± 0.37	0.11 ± 0.52	0.10 ± 0.49
Sperm progressive motility (%)	17.5 ± 2.61	19.1 ± 2.23	21.2 ± 2.11
Sperm with normal morphology (%)	15.83 ± 4.61	17.02 ± 4.32	16.45 ± 4.69
Teratzoospermia index (%)	1.62 ± 0.12	1.59 ± 0.21	1.60 ± 0.34

*SHBG* Sex hormone-binding globulin, *FSH* Follicle stimulating hormone, *LH* Luteinizing hormone, *MOSH* Male Obesity Secondary Hypogonadism. Statistical test used: Wilcoxon test was used to identify the significance between continuous preoperative values and follow-up data. Data Values are expressed as mean + SD or N (%), * *p* < 0.05 compared to pre-surgical values. ** *p* < 0.01 compared to pre-surgical values

**Table 3:** Comparison between preoperative sexual function and follow-up data at 12- and 18-months post- Sleeve Gastrectomy

	Pre-operative	12 Months	18 Months
Sexual desire	9.67 ± 1.04	15.5 ± 1.16 *	18.9 ± 0.32 **
Erectile function	22.73 ± 7.41	27.28 ± 6.12 *	27.11 ± 4.21*
Orgasmic function	9.75 ± 3.82	10.68 ± 4.11	11.20 ± 2.02
Intercourse satisfaction	6.65 ± 1.67	8.39 ± .81	8.81 ± .92*
Overall satisfaction	6.26 ± 2.11	8.53 ± 1.1	8.60 ± 42*

IIEF questionnaire was used for sexual function assessment. Statistical test used: Wilcoxon test was used to identify the significance between continuous preoperative values and follow-up data. Values are expressed as mean + SD, * *p* < 0.05 compared to pre-surgical values. ** *p* < 0.01 compared to pre-surgical values

## Discussion

Obesity impairs male fertility and can be explained by a variety of factors, including altered sexual health, endocrine disturbances, accumulation of environmental toxins in adipose tissue, elevated scrotal temperatures, and genetic abnormalities leading to abnormal spermatogenesis. Human body mass is a fundamental determinant of systemic low-grade inflammation levels that could be tailored after weight loss. These dramatic changes are illustrated by the regulation of 51 inflammation-associated proteins, obviously c-reactive protein (CRP) [16]. Recent studies have highlighted the impact of obesity and bariatric surgery on sperm parameters [2, 17]. Published data suggest that bariatric surgery can improve multiple obesity-related comorbidities, including cardiovascular disease, diabetes, and hypertension, and significantly reduce medication use and costs [18]. Reducing fat mass after weight loss can reduce fat accumulation in the thigh and suprapubic region, lower testicular temperature, and thus promote spermatogenesis [19]. Our study demonstrates that weight loss achieved after gastric sleeve surgery increases total testosterone, free testosterone, and SHBG, thereby completely resolving MOSH in severely obese men. Our results show that sexual function improves with increasing Sperm count. We did not see any significant improvement in sperm motility and morphology over time. Our study showed that 33.3% of severely obese men who underwent sleeve gastric surgery had preoperative testosterone concentrations in the hypogonadal range (< 9.2 nmol/L) [13]. After surgery, all hypogonadal patients recovered, and total and free testosterone concentrations increased significantly across the group, suggesting an improvement in the hormonal pattern characteristic of MOSH. The etiology of MOSH is likely multifactorial, including reduced levels of SHBG in obesity, increased aromatase activity, and production of adipocytokines and gut-derived endotoxins that impair kisspeptin signaling in the hypothalamus (and thus GnRH secretion) [20]. The present studies show that SHBG concentrations and free testosterone concentrations increase after gastric sleeve surgery, emphasizing that not all observed changes in testosterone concentrations are caused by the effect of obesity and weight loss on SHBG concentrations. Our results are consistent with data from previous studies analyzing the effect of bariatric surgery on testosterone levels in men [21]. Our study assessed sexual function using the IIEF questionnaire, which assessed erectile function, orgasmic function, libido, intercourse satisfaction, and overall satisfaction [14]. Our results showed that gastric sleeve surgery improved erectile function, libido, and satisfaction without significantly affecting orgasmic function. The effect of bariatric surgery on erectile function has been much less studied than its effect on hormonal alertness. Nonetheless, our data are consistent with many previous studies confirming the positive effect of bariatric surgery on IIEF [22]. After sleeve gastrectomy, we observed an increase in sperm alertness in the cases studied, which was similar to the increase in testosterone alertness and sexual function during follow-up but had no effect on other semen parameters. This finding is interesting considering the significant improvement in hormonal status associated with roundness in secondary hypogonadism. Many previous studies have examined the benefits of substantial weight loss after bariatric surgery. Initially, several case series focused again on the detrimental effects of roundness surgery on male fertility [23, 24]. These anecdotal cases have been followed by a number of opposing prospective studies involving small numbers of subjects. El Bardisi et al. [2] examined sleeve gastrectomy results for semen parameters 12 months after the procedure and found improved sperm vigilance in men with previous oligospermia and azoospermia. Samavat et al. [25] also noted improvements in semen volume and motility 6 months after surgery. Legro et al. [17] and Reis et al. [26] independently found no change in sperm parameters at 12 and 24 months after surgery. Paradoxically, two recent studies reported a decrease in sperm alertness and total sperm number [27, 28]. Carette et al. [27] found a decrease in the number of morphologically normal spermatozoa 12 months after roundness surgery. A recent meta-analysis summarized the existing literature on the effects of bariatric surgery on sperm characteristics. It concluded that it did not affect sperm volume, alertness, total number, morphology, motility or viability [29]. Another study by Inka et al. [30] showed no effect of bariatric surgery on sperm count or sperm attention at any time during the study period. Likewise, there is little information on the effect of Diet-Induced Weight Loss on sperm parameters. However, it appeared to improve some parameters of semen quality, such as sperm alertness, total sperm number, or sperm morphology [7]. It could be augmented by regular physical exercise as the latter was reported to improve cardiorespiratory fitness, emotional well-being, level of glycated hemoglobin, and insulin sensitivity [31]. However, due to the small number of studies available and the lack of direct comparisons between life-saving interventions and circular surgery, this information should be interpreted with caution. We performed this prospective study because of inconsistent results from the above-mentioned initial report. We dissected sexual function, semen parameters, and hormonal biographies of infertile fat cases before and after gastric sleeve surgery. Overall, cases witnessing sleeve gastrectomy significantly improved their serum testosterone levels, which is consistent with previous reports (El Bardisi H. etal., [2] and SamavatJ. etal [25]). There was a trend toward improvement in all sperm parameters postoperatively; however, only sperm count was statistically significant (Table 2). Most of the cases in previous studies had normal mean semen parameters, and our study is probably the first to stratify cases of infertile adipose tissue with abnormal preoperative semen analysis parameters and estimate their semen analysis, sexual function and hormonal status Possibilities to improve after gastrectomy surgery with a fairly long postoperative follow-up (18 months). Obese cases with oligospermia before surgery had a statistically significant improvement in sperm vigilance at 12 and 18 months after surgery. Increases in serum testosterone were consistently observed in all cases (Table 2). Differences in bariatric surgery reports on sperm parameters between this work and previous reports can be explained by differences in study sample size and follow-up times. Previous studies were case series with shorter follow-up periods, which are less preferred for reporting surgical outcomes and cannot draw definitive conclusions. Sermondade et al. [24], for example, evaluated their patients too soon after surgery (3 months) during rapid weight loss. In terms of sexual dysfunction, the underlying mechanisms may be multifactorial, including hypogonadism and the associated metabolic syndrome, which has been linked to endothelial and erectile dysfunction [32]. As a result, the mechanisms underlying improved sexual function are closely linked to an improvement in the hormonal and metabolic milieu following weight loss. Our study’s limitations include its observational nature and the absence of more than one semen sample at each time in each patient. The main strength of this study is that it is prospective, with a sufficient number of patients, and that data is collected throughout the acute weight loss phase and until 18 months after surgery (when weight often stabilizes). This provides critical information about the potential impact of active or stabilized weight loss on the parameters under consideration. Furthermore, we carefully selected patients to rule out any confounders that might influence gonadal function.

## Conclusion

Post-sleeve gastrectomy weight loss significantly improves testosterone deficiency, sexual performance, and Sperm count in obese infertile men. Further large-scale studies are demanded to consolidate the findings handed by the present study.

## Data Availability

Data sets used in this study are available upon reasonable request from the corresponding authors.

## Abbreviations

BMI: Body mass index
HDLc: High density lipoprotein cholesterol
LDLc: Low density lipoprotein cholesterol
%TWL: Percent total weight loss
SHBG: Sex hormone-binding globulin,
FSH: Follicle-stimulating hormone
LH: Luteinizing hormone
CMIA: The chemiluminescent microparticle immunoassay
IIEF: International Index of Erectile Function questionnaire
OSA: Obstructive sleep apnea
MOSH: Male obesity secondary hypogonadism
